# Development and Characterization of Functional Gummies Enriched with País Grape Extract for Presbyphagia

**DOI:** 10.3390/gels12080674

**Published:** 2026-07-28

**Authors:** Peña-Portillo Glenda-Caridad, Bastías-Montes José-Miguel, García-Flores Virginia-Andrea, Acuña-Nelson Sergio-Miguel

**Affiliations:** 1 Departamento de Ingeniería en Alimentos, Universidad del Bío-Bío, Avenida Andrés Bello 720, Chillán 3780000, Chile; glenda.pena2201@alumnos.ubiobio.cl (P.-P.G.-C.); jobastias@ubiobio.cl (B.-M.J.-M.); 2 Departamento de Ciencias de la Rehabilitación en Salud, Universidad del Bío-Bío, Avenida Andrés Bello 720, Chillán 3780000, Chile; vgarcia@ubiobio.cl

**Keywords:** dysphagia, texture-modified foods, grape pomace, NaDES, functional gummies, antioxidants

## Abstract

The increasing prevalence of presbyphagia and mild dysphagia among adults over 65 years (International Dysphagia Diet Standardisation Initiative, IDDSI, levels 4–6) has increased the demand for texture-modified foods that ensure safe swallowing while providing added health benefits. This study aimed to develop functional gummy-like systems containing grape pomace extracts obtained by ultrasound-assisted extraction using either a natural deep eutectic solvent (NaDES; choline chloride:citric acid, 1:1) or water, and to evaluate the effects of extraction medium and formulation composition on their physicochemical, textural, and bioactive properties. A simplex-centroid mixture design was used to assess the combined effects of gelatin (4–8%), low-methoxyl pectin (1–3%), and extract concentration (5–10%). NaDES-based formulations exhibited lower water activity and a more homogeneous moisture distribution than aqueous counterparts, indicating improved water structuring within the gel network. Intermediate gelatin–pectin ratios produced cohesive matrices with moderate firmness, consistent with IDDSI levels 5–6. Formulation F5 (6% gelatin, 1% pectin, 7.5% extract) showed the best overall performance, combining favorable textural properties with the highest total polyphenol content and antioxidant activity. These findings support the use of NaDES-extracted grape pomace for developing functional foods suitable for individuals with dysphagia.

## 1. Introduction

Population aging is one of the most significant demographic trends worldwide and is associated with an increased prevalence of age-related physiological changes that affect nutritional status and food intake. Among these changes, alterations in the oral, pharyngeal, and esophageal phases of swallowing may compromise eating efficiency and safety, potentially increasing the risk of malnutrition, dehydration, and reduced quality of life [1,2]. Age-related modifications in swallowing function are commonly described as presbyphagia, a term referring to the characteristic physiological adaptations that occur during healthy aging. In contrast, dysphagia refers to a clinically significant swallowing disorder that impairs the safe and effective transport of food or liquids from the mouth to the stomach. Although presbyphagia does not necessarily imply pathology, it may reduce functional reserve and increase susceptibility to swallowing difficulties under conditions of illness, frailty, or neurological impairment [3,4,5].

The growing prevalence of swallowing-related disorders among older adults has stimulated the development of texture-modified foods designed to facilitate oral processing and swallowing. Conventional texture-modified products include purees, thickened beverages, soft gels, and semi-solid formulations. Although these products may improve food manageability, they often exhibit limited sensory appeal, poor portability, or reduced consumer acceptance [6,7]. Consequently, there is increasing interest in developing alternative food matrices capable of combining desirable textural characteristics with the incorporation of functional ingredients.

Gummy-type matrices have emerged as promising delivery systems for bioactive compounds due to their ease of consumption, formulation flexibility, and capacity to incorporate nutritionally relevant ingredients. The physicochemical and textural characteristics of these systems can be modulated through the selection and proportion of hydrocolloids, allowing the development of products with distinct structural and mechanical properties [7,8]. Among the hydrocolloids commonly used in gummy formulations, gelatin and pectin are particularly attractive because of their complementary gelling mechanisms and widespread use in food applications. The interaction between these biopolymers may influence water retention, gel strength, elasticity, and overall product stability, making hydrocolloid composition a critical factor in formulation design [8,9,10].

Simultaneously, there is increasing interest in the valorization of agro-industrial by-products as sustainable sources of bioactive compounds. Grape pomace, the principal solid residue generated during winemaking, contains substantial amounts of phenolic compounds, including flavonoids, phenolic acids, tannins, and anthocyanins, which are recognized for their antioxidant properties [11]. The recovery and incorporation of these compounds into food products not only contributes to circular economy strategies but also provides opportunities for the development of value-added functional ingredients.

The efficiency of bioactive compound recovery is strongly influenced by extraction methodology. In recent years, natural deep eutectic solvents (NaDES) have gained attention as environmentally friendly extraction media capable of improving the recovery of phenolic compounds from plant materials [12]. These solvents are typically composed of naturally occurring and food-compatible constituents that exhibit low toxicity, biodegradability, and favorable extraction performance. Several studies have reported enhanced extraction yields and improved preservation of phenolic compounds when NaDES systems are combined with emerging extraction technologies such as ultrasound-assisted extraction (UAE). Beyond extraction efficiency, the solvent employed may also influence the physicochemical characteristics of the recovered phytochemicals, including their molecular interactions, solubility, and stability, which may subsequently affect their behavior after incorporation into food matrices. Recent evidence suggests that the polarity and hydrogen-bonding capacity of NaDES can modify interactions between phenolic compounds and food biopolymers, potentially influencing the structural organization, water retention, and textural properties of hydrocolloid-based systems. Furthermore, because NaDES components may remain in the final extract, these systems present particular interest for food applications where solvent compatibility is essential. Nevertheless, little information is available regarding how the extraction medium itself influences the technological performance of gelatin–pectin gummy matrices, providing an additional rationale for the present study [13,14,15].

Despite the growing body of literature on grape pomace valorization, functional gummy formulations, and NaDES-assisted extraction, important knowledge gaps remain. Most previous studies have focused either on optimizing the extraction of phenolic compounds or on developing functional gummy formulations independently. Consequently, little information is available on how the extraction medium itself influences the technological performance of hydrocolloid-based gummy matrices after incorporation of the extract. In particular, the combined effect of aqueous and NaDES-derived País grape pomace extracts and gelatin–pectin composition on moisture-related properties, texture, color, antioxidant capacity, and bioactive compound retention has not been systematically investigated. Understanding these interactions is essential for designing functional texture-modified foods in which both the extraction strategy and the gel network contribute to product quality, stability, and potential applicability for the management of swallowing disorders [16,17,18]. Therefore, this study addresses an important gap by integrating extraction technology with hydrocolloid formulation in a single experimental approach.

Based on this rationale, the objective of the present study was to evaluate the combined influence of the extraction medium (aqueous versus NaDES extract) and the gelatin–pectin ratio on the physicochemical, textural, and antioxidant properties of gummy-type matrices enriched with País grape pomace extracts. Specifically, this work aimed to (i) compare the retention of bioactive compounds obtained through different extraction media, (ii) assess the effect of hydrocolloid composition on matrix properties, and (iii) identify formulations exhibiting the most balanced combination of physicochemical, textural, and bioactive attributes through multivariate analysis.

## 2. Results and Discussion

### 2.1. Physicochemical Properties

Although water activity (a_w_) is traditionally associated with microbiological stability, it plays a decisive structural role in hydrocolloid-based gelled matrices. In polymeric systems, water acts as a plasticizer that modulates molecular mobility and the density of the three-dimensional network [19]. In this regard, the a_w_ should be interpreted as both an indicator of stability and a structural variable that determines the system’s mechanical behavior.

**Figure 1:**
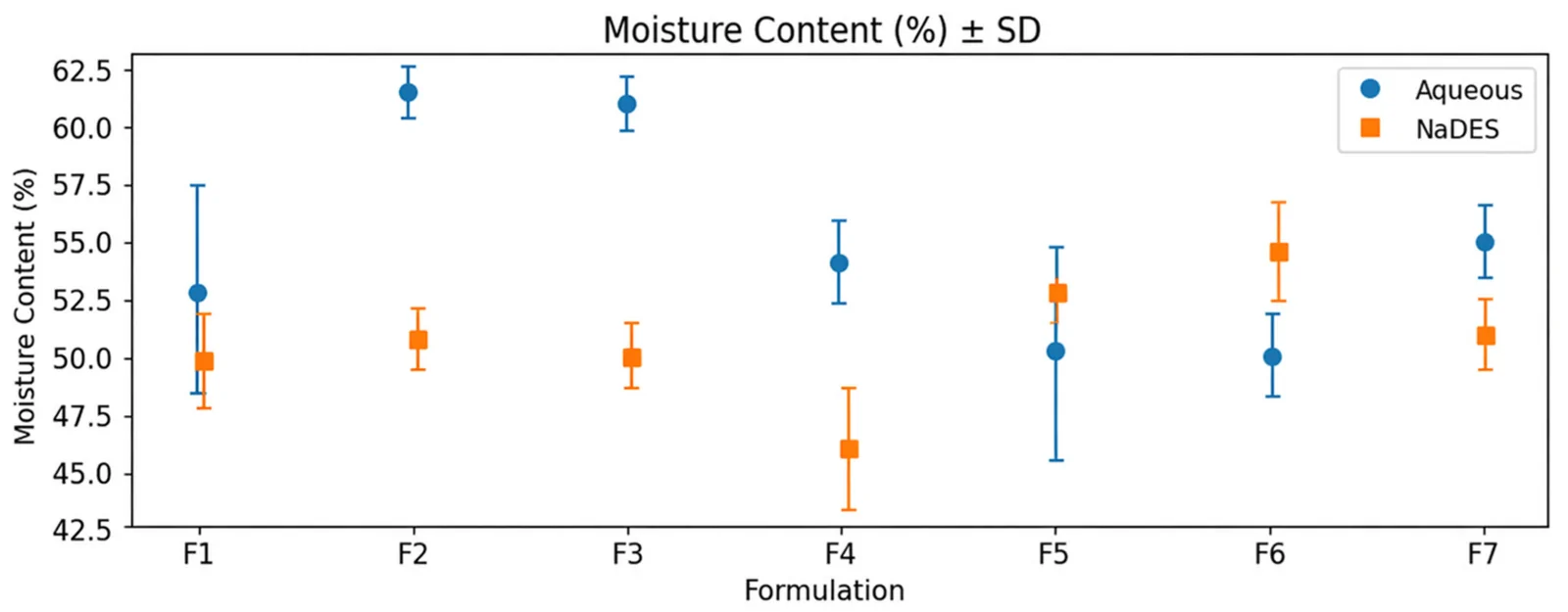
Comparison of moisture content (%) among formulations (F1–F7) produced with aqueous and NaDES grape pomace extracts (mean ± SD).

Figure 1 compares the moisture content of the seven formulations (F1–F7) prepared with aqueous extracts and their corresponding counterparts prepared with NaDES extracts. Formulations containing the aqueous extract exhibited higher and more variable moisture contents, reaching values of up to 62.05%, whereas those prepared with the ChCl acid NaDES extract showed consistently lower moisture values (approximately 50–55%). These results indicate that the extraction medium influenced the final moisture content of the gummy matrices. A plausible explanation is that the extensive hydrogen-bonding network established by the choline chloride–citric acid NaDES, together with its interactions with gelatin and pectin, modified the organization of the gel network and the distribution of water within the matrix. Consequently, less water may have remained as free water, leading to lower and more uniform moisture contents in the NaDES formulations. However, confirmation of changes in water mobility or the relative proportions of free and bound water would require complementary analyses beyond moisture determination.

**Figure 2:**
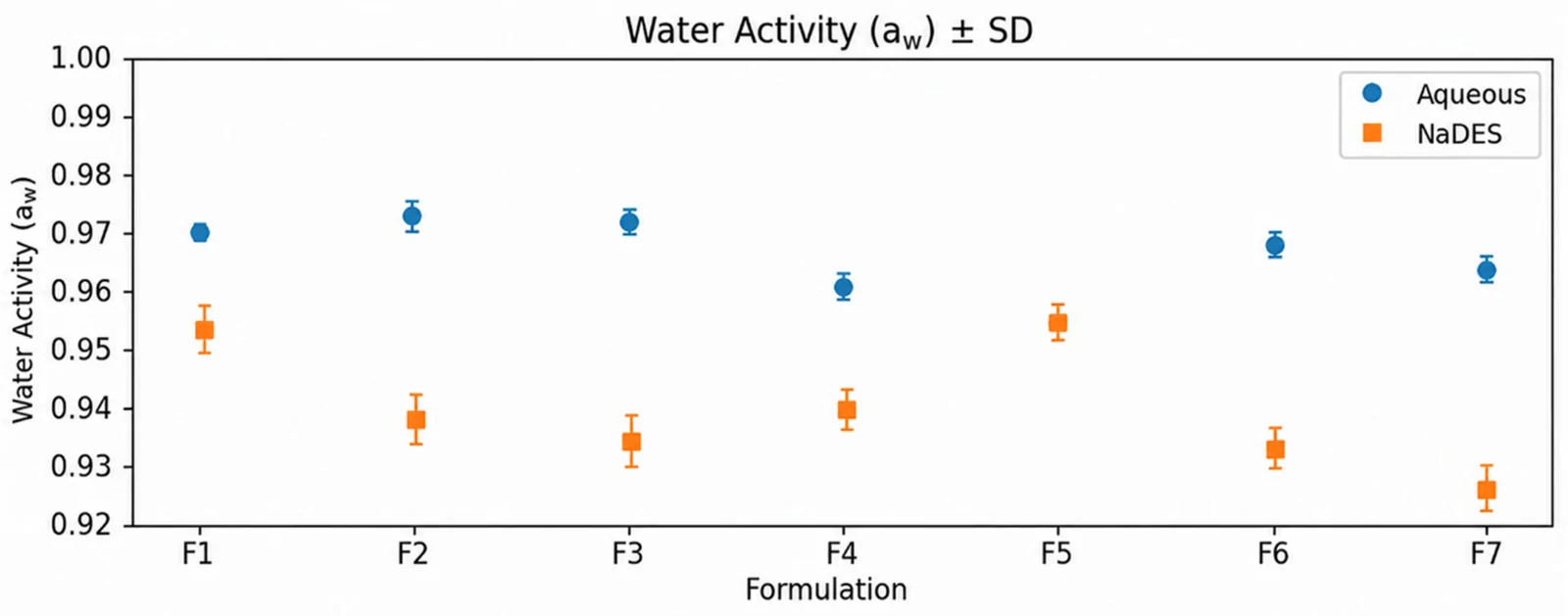
Comparison of water activity (a_w_) among formulations (F1–F7) produced with aqueous and NaDES grape pomace extracts (mean ± SD).

However, analyzing Figure 2, which shows the a_w_ values, reveals that the relationship between total moisture and water activity is not proportional. Both series exhibit high a_w_ values (>0.90), but the NaDES system consistently shows lower values (0.925–0.954) than the aqueous system (0.9538–0.97285). This decoupling of total water content and a_w_ has been widely described in concentrated systems where solute-water interactions reduce the thermodynamically available fraction [20,21].

The traditional a_w_ value range for gummy-type confectionery is from 0.75 to 0.85 [22,23]. Therefore, the values observed in both experimental series are considerably higher, indicating a highly plasticized matrix. However, the lower a_w_ in the NaDES formulations, as shown in Figure 2, suggests a structuring effect associated with extensive hydrogen bond networks formed between the eutectic components and water. This reduces their effective mobility while maintaining high moisture content.

As water availability increases, as observed in Figure 2, where the aqueous formulations have higher a_w_ values, mechanical strength decreases. This confirms the role of water in amorphous matrices affecting structural properties. This phenomenon is also associated with decreased structural cohesion of the network due to reconfigured hydrogen bonds between water and biopolymers, resulting in weaker and less rigid matrices [24,25]. Thus, a side-by-side comparison of Figure 1 and Figure 2 reveals that the physicochemical state of the water, rather than absolute humidity, is the determining factor in the mechanical response.

In dysphagia-related applications, this hydration modulation takes on functional significance. A rigid matrix increases the load on the tongue and the effort required for oral processing [26], while a matrix with excessively high a_w_ can compromise structural cohesion. However, the IDDSI framework establishes that clinical classification is based on functional tests rather than isolated instrumental parameters [27]; therefore, a_w_ should be interpreted as an indirect structural modulator.

Taken together, a comparative analysis of Figure 1 and Figure 2 shows that the NaDES system reduces water activity and stabilizes moisture content across formulations. This dual effect, lower water activity (a_w_) and greater moisture consistency, suggests a more efficient organization of water within the matrix. This could explain the more balanced mechanical behavior observed in formulations such as F5.

### 2.2. Color

Analysis in the CIELAB color space revealed consistent chromatic differences between formulations, confirming that the matrix type and extraction medium directly influence the system’s optical properties. The CIELAB model interprets variations in perceptual terms: L* represents lightness, a* represents the red–green dimension, and b* represents the yellow–blue dimension. It is a widely accepted system for evaluating color differences in food matrices [28,29].

The statistical analysis (two-way ANOVA, *p* < 0.05) indicated that the extraction method (NaDES vs. aqueous), the formulation (F1–F7), and their interaction significantly affected L*, a*, b*, and values, as shown in Table 1. These results confirm that color development is not solely dependent on formulation composition, but also on the extraction environment of the bioactive compounds.

Regarding lightness (L*), NaDES-based gummies showed values ranging from 27.23 to 32.61, while aqueous extracts ranged from 24.93 to 32.12. In general, NaDES formulations maintained slightly higher L* values, particularly in F2 and F4, suggesting a higher optical brightness in these systems. The reduction in L* observed in certain formulations (e.g., F7 in both systems) indicates increased light absorption, which is commonly associated with higher concentrations of chromophoric compounds or stronger pigment–matrix interactions. This behavior is consistent with reports showing that phenolic-rich extracts and betalain-like pigments can decrease lightness due to increased optical density of the matrix [30,31,32].

Significant differences were also observed in the a* coordinate, which represents redness intensity. NaDES formulations exhibited markedly higher a* values (6.54–12.99) compared to aqueous formulations (5.84–8.93). The most pronounced difference was observed in F3, where NaDES extraction yielded a substantially higher redness (12.40 ± 2.90) than its aqueous counterpart (8.93 ± 0.79). This suggests that NaDES systems enhance either the extraction efficiency or the stabilization of red chromophores, reducing pigment degradation during processing. Natural deep eutectic solvents have been widely reported to improve solubilization and stability of phenolic compounds through strong hydrogen bonding interactions, which may explain the enhanced chromatic expression observed in this study [33,34,35].

For the b* parameter, NaDES formulations generally exhibited higher values (3.73–7.04) compared to aqueous systems (1.46–4.63), indicating a greater contribution of yellow tones. The most pronounced difference was observed in F4, where NaDES (7.04 ± 1.21) nearly doubled the b* value of the aqueous system (2.47 ± 0.51). This suggests that the NaDES medium preserves or enhances compounds responsible for yellow coloration or limits their degradation during thermal processing. In sugar-rich matrices, variations in b* may also be influenced by Maillard-type reactions and pigment transformation pathways, which alter the balance between yellow and blue components [28,36].

The chromaticity values (C*ab), calculated from the a and b coordinates, provided additional information on color saturation and intensity, as shown in Table 1. In the NaDES system, the chromaticity values ranged from 8.75 (F1) to 13.54 (F5), with formulations F3 (13.09), F4 (12.98), and F5 (13.54) exhibiting the highest saturation. In contrast, the gummy candies made with aqueous extracts showed lower chromaticity values, ranging from 6.07 (F2) to 9.53 (F7). Particularly noteworthy were formulations F2 and F4, whose chroma values decreased from 12.22 to 6.07 and from 12.98 to 7.05, respectively, when the NaDES extract was replaced by the aqueous extract. These differences indicate that the effect of the extraction medium was formulation-dependent and suggest that the interaction between the extracted pigments and the gummy matrix influenced the final color saturation. Since chroma reflects the vividness or saturation of the perceived color, the consistently higher C*ab values observed in the NaDES formulations indicate a more intense visual appearance. This trend is consistent with the higher a* and b* coordinates measured for the same formulations and agrees with reports highlighting that the concentration and stability of natural pigments, particularly betalains and other phenolic chromophores, are major determinants of color saturation and visual quality in food products [33,34,35].

The higher chroma values observed in the NaDES system were also accompanied by greater ΔE values, indicating that the enhanced saturation translated into larger perceptible color differences relative to the reference. Together, C*ab and ΔE demonstrate that the extraction medium influenced not only the hue coordinates but also the overall visual impact of the gummy formulations.

The total color difference (ΔE) confirmed clear perceptible differences across all formulations. NaDES-based gummies exhibited ΔE values ranging from 29.71 ± 0.28 to 34.92 ± 2.52, while aqueous formulations ranged from 26.03 ± 0.91 to 32.69 ± 0.71. According to established thresholds, ΔE values above 2–3 are easily perceptible by the human eye under controlled condition [28], indicating that all formulations displayed clearly distinguishable color differences relative to the reference system. Importantly, NaDES formulations generally produced higher ΔE values than aqueous extracts, particularly in F2, F4, and F5, suggesting that NaDES extraction enhances overall chromatic intensity.

The interaction between extraction medium and formulation was particularly relevant for ΔE, as certain formulations (e.g., F5 and F6) showed pronounced differences between NaDES and aqueous systems. This indicates that the effect of the extraction solvent is not uniform across formulations and depends on the specific matrix composition, likely due to differences in pigment binding, solubility, and gel network interactions.

Overall, the results demonstrate that NaDES extraction leads to gummies with enhanced chromatic expression, characterized by higher redness (a*), increased yellowness (b*), and greater overall color difference (ΔE) compared to aqueous extracts. These findings suggest that NaDES not only improves extraction efficiency but also contributes to the stabilization of chromophoric compounds within the gummy matrix, resulting in more intense and visually distinct products. Conversely, aqueous extracts tend to produce slightly lower chromatic intensity, although in some formulations they may favor more moderate color development depending on matrix interactions.

These observations are consistent with previous studies reporting that natural deep eutectic solvents enhance the recovery and stability of plant pigments through strong intermolecular interactions and improved solvation properties compared to conventional aqueous systems [33,34,35]. Therefore, the use of NaDES represents a promising strategy to modulate and enhance color properties in gummy candy formulations, with potential implications for product design and consumer perception.

### 2.3. Texture Profile Analysis of Gummy-Type Products Formulations

**Figure 3:**
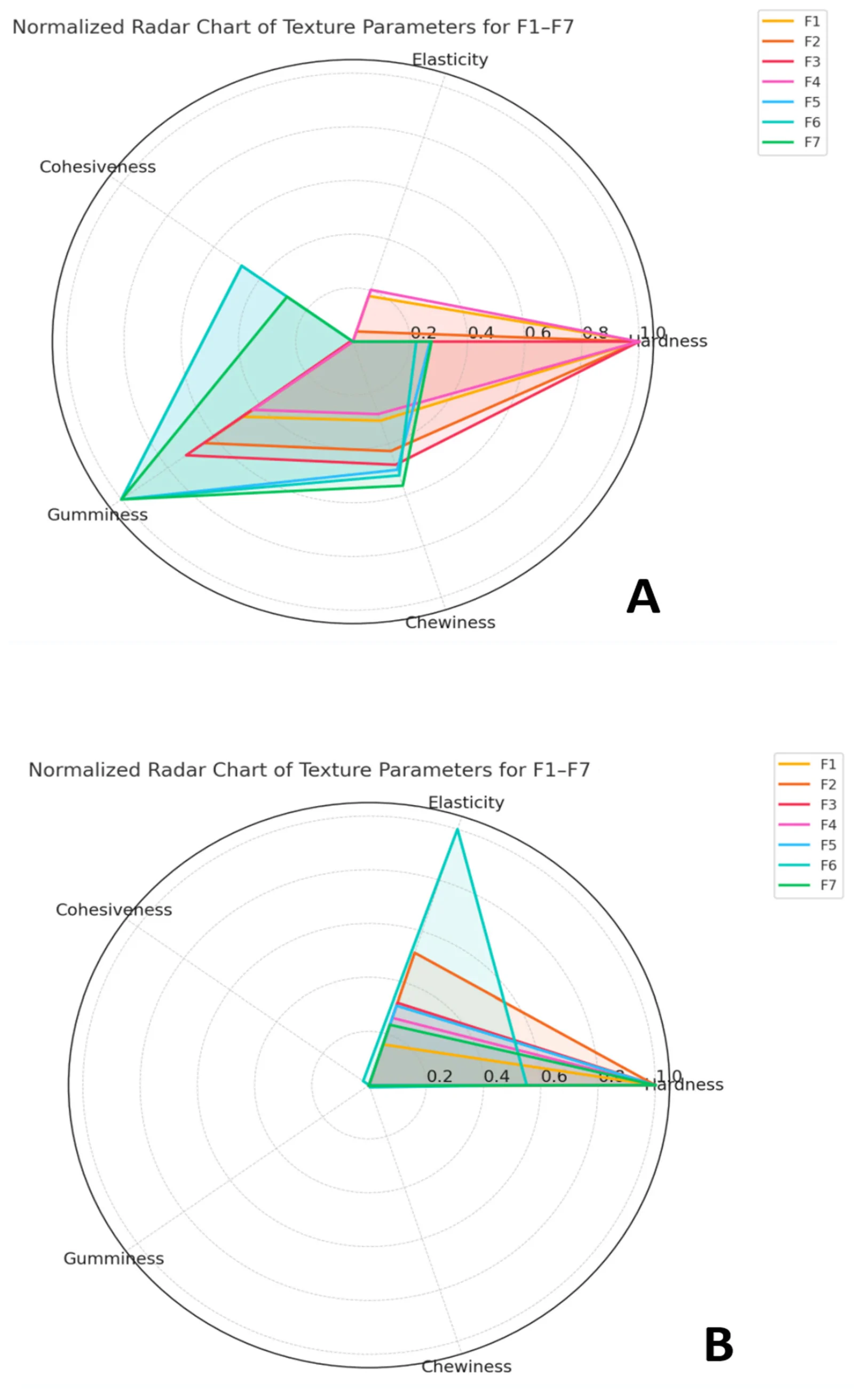
Comparative analysis of the texture profiles of formulations F1–F7. ((**A**) Formulations with NaDES-based extract. (**B**) Formulations with aqueous extract) using a normalized radial plot.

Figure 3A,B present normalized radar plots illustrating the textural profiles of gummy-like matrices prepared with NaDES and aqueous País grape extracts, respectively. Although both systems were mechanically characterized, the discussion focuses primarily on the NaDES formulations because they represent the functional delivery system developed in this study. Texture profile analysis (TPA) revealed marked differences among formulations, particularly in hardness, cohesiveness, adhesiveness, gumminess, and chewiness, whereas springiness exhibited comparatively limited variation across the experimental design.

Food oral processing is a rhythmic, adaptive neurosensorimotor process involving incision, crushing, mastication, and salivation to produce a cohesive, lubricated bolus suitable for swallowing. For older adults with presbyphagia or mild dysphagia, the mechanical properties of food are particularly important because bolus formation, oral processing efficiency, and swallowing safety are directly influenced by the structural integrity and deformation behavior of the food matrix [37,38]. Therefore, developing gummy matrices requires balancing sufficient mechanical strength to maintain structural integrity with adequate deformability to minimize chewing effort while ensuring safe bolus formation.

The textural differences observed among formulations reflected the combined influence of gelatin, pectin, and extract concentration on gel network formation. Gelatin was the primary structural component governing the mechanical strength of the matrices [39]. This behavior is consistent with the gelation mechanism of protein-based food gels, where increasing polymer concentration increases junction-zone density and mechanical resistance [40,41]. As gelatin concentration increased, hardness, gumminess, and chewiness generally increased, reflecting the formation of a denser three-dimensional protein network stabilized by triple-helix junction zones. Such reinforcement enhances resistance to compression but also increases the mechanical work required during mastication. Excessively high hardness and chewiness may prolong oral processing time and increase masticatory effort, particularly in older adults with reduced bite force or compromised oral function [31,34,35,37].

Pectin, although incorporated at lower concentrations, played a complementary role by reinforcing the continuity and stability of the mixed hydrocolloid network. The interaction between gelatin and citrus pectin likely promoted a more integrated gel structure through hydrogen bonding and water immobilization, resulting in increased cohesiveness without substantially increasing elasticity. Cohesiveness, defined as the ability of a sample to withstand a second deformation relative to the first, reflects the strength of the internal bonds within the gel network and is essential for maintaining bolus integrity during oral processing. A cohesive bolus is less prone to premature fragmentation, thereby facilitating safer swallowing [38,42,43,44].

The concentration of País grape extract also contributed to the mechanical behavior of the matrices. Besides increasing the aqueous phase, phenolic compounds can interact with both gelatin and pectin through hydrogen bonding and hydrophobic interactions, modifying polymer–polymer associations within the gel network. These interactions may either reinforce or partially interfere with hydrocolloid cross-linking depending on the relative concentrations of the biopolymers and phenolic compounds, thereby influencing the balance between rigidity and deformability.

Adhesiveness, representing the force required to detach the gummy surface from oral or instrumental surfaces, is another important quality attribute for texture-modified foods. Excessively adhesive products may remain attached to the palate, tongue, or teeth, increasing oral residue and potentially compromising swallowing safety in individuals with impaired oral clearance. This characteristic has been recognized as an important determinant of oral clearance efficiency and swallowing safety in texture-modified foods [45,46]. Conversely, matrices exhibiting moderate adhesiveness are generally considered more suitable because they maintain sufficient surface interaction for cohesive bolus formation without causing undesirable stickiness during oral processing. In the present study, adhesiveness remained within a moderate range across the optimized formulations, indicating that increasing hydrocolloid concentration reinforced the internal gel network without producing excessive surface tackiness.

Beyond formulation composition, the extraction medium also influenced the mechanical behavior of the gels. Compared with aqueous extracts, NaDES-containing formulations consistently exhibited greater cohesiveness, gumminess, and chewiness, suggesting that the solvent environment modified intermolecular interactions within the hydrocolloid network. The unique polarity and hydrogen-bonding capacity of NaDES may enhance interactions among gelatin, pectin, and phenolic compounds, producing a more interconnected three-dimensional structure than that obtained with conventional aqueous extracts [32,33,47]. Radar plot visualization clearly illustrates this structural amplification, particularly in formulations containing intermediate to high hydrocolloid concentrations.

Among all formulations, F5 exhibited the most balanced textural profile. Rather than maximizing mechanical resistance, this formulation combined moderate hardness with high cohesiveness, controlled adhesiveness, intermediate gumminess, and acceptable chewiness. This combination suggests a gel network capable of maintaining structural integrity during handling while remaining sufficiently deformable to facilitate bolus formation with relatively low oral effort. These characteristics are particularly desirable for older adults, in whom both excessive firmness and excessive stickiness may negatively affect oral processing efficiency. The central position of F5 within the PCA biplot (Section 2.5) further supports this interpretation by demonstrating simultaneous association with favorable physicochemical, antioxidant, and textural attributes.

In contrast, F7 exhibited the highest mechanical resistance, with the greatest hardness, cohesiveness, gumminess, and chewiness values among all formulations. Although this formulation demonstrates the strongest gel network, its elevated mechanical strength would likely require greater masticatory effort and longer oral processing time before bolus formation. Previous studies have shown that highly gummy and cohesive hydrocolloid-based foods may increase oral workload and contribute to masticatory fatigue in older adults with reduced chewing performance [31,34,37,48]. Therefore, maximizing mechanical strength alone does not necessarily improve suitability for populations with compromised oral function.

Overall, these results demonstrate that the texture of functional gummy matrices is governed by the synergistic effects of gelatin, pectin, extract concentration, and extraction medium. While gelatin primarily determines mechanical resistance, pectin contributes to network cohesion and structural stability, the grape extract modulates hydrocolloid interactions, and the NaDES system further reinforces the gel architecture through enhanced intermolecular interactions. Within this multicomponent system, formulation F5 (6% gelatin, 1% pectin, and 7.5% NaDES extract) achieved the most appropriate compromise between structural integrity, controlled adhesiveness, mechanical resistance, and ease of oral breakdown, while simultaneously providing approximately 89 mg GAE/100 g of total phenolic compounds. This balance supports its potential application as a functional gummy matrix specifically designed for older adults with age-related alterations in oral processing.

### 2.4. Bioactive Properties

#### 2.4.1. Total Polyphenol Content (TPC)

The total polyphenol content (TPC) of the gummy-like matrices made with grape pomace extract, obtained via ultrasound-assisted extraction with a natural eutectic solvent (NaDES) of choline chloride and citric acid (1:1), ranged from 58.04 to 89.43 mg GAE/100 g. These results represent an overall retention of 6–9% relative to the original extract (approximately 1000 mg GAE/100 g), which is reasonable considering the susceptibility of phenolic compounds to heat and interaction with protein-polysaccharide matrices.

Formulation F5 (6% gelatin, 1% pectin, and 7.5% extract) exhibited the highest TPC (89.43 mg GAE/100 g), followed by formulations F6 and F7 (83.41 and 77.39 mg GAE/100 g, respectively). This suggests that an intermediate level of gelatin (6%) combined with a low proportion of pectin (1–2%) favors polyphenol retention. This can be attributed to the appropriate balance between the protein (gelatin) and polysaccharide (pectin) networks, which allows for the partial encapsulation of phenolic compounds without excessive gelation or syneresis.

In particular, the fact that formulations F5 and F6 (intermediate G:P ratios and 7.5% extract) exhibited the highest TPC is consistent with what has been reported by [11], who identified a synergy between gelatin and pectin that optimizes the microstructure of the gel and protects phenolic compounds.

Several authors have reported that the ratio of gelatin to pectin significantly influences the stability and release of bioactive compounds. For instance, recent studies indicate that high pectin ratios increase cross-linking density and restrict antioxidant diffusion during cooling [10]. A gelatin-dominated matrix tends to better retain hydrophilic phenolic compounds through noncovalent interactions, such as hydrogen bonds between phenolic hydroxyl groups and the carbonyl or amide groups of proteins. These interactions, as well as hydrophobic and van der Waals interactions, stabilize the polyphenols within the protein network [49,50,51].

Furthermore, using the NaDES solvent (choline and citric acid) appears to have facilitated the extraction of a thermally stable polyphenolic fraction. This type of solvent has been associated with higher levels of phenolic acids and glycosylated flavonols, which are more compatible with food matrices and undergo less oxidative degradation during thermal processing [52]. Therefore, although the final content is much lower than that of the original extract, the relative retention efficiency is high compared to that of traditional aqueous systems, which typically retain less than 5% of the TPC.

In terms of trends, formulations with a higher proportion of pectin (F2 and F7) tend to have intermediate to high total phenolic content (TPC) values, which indicates a synergistic effect between gelation and polyphenol retention. This could be due to an interaction between the pectin’s carboxyl groups and the polyphenols’ hydroxyl groups. This interaction partially stabilizes the compounds during storage but may also limit their analytical extractability.

The observed distribution suggests that a structural balance of 6% gelatin, 1–2% pectin, and 7.5% extract (F5–F6) provides optimal phenolic retention without compromising network integrity.

#### 2.4.2. Total Anthocyanin Content (TAC)

The total anthocyanin content (TAC) of the gummy matrices made with the NaDES (choline chloride-citric acid, 1:1) extract from Uva País grape pomace ranged from 0.100 to 0.367 mg C3G/100 g. The highest value was recorded in formulation F7, and the lowest value was recorded in formulation F4. In contrast, the estimated TAC values for matrices prepared with an aqueous extract ranged from 0.055 to 0.202 mg C3G/100 g. Given that anthocyanins are inherently unstable molecules and prone to degradation due to environmental exposure, gastrointestinal conditions, and fluctuations in pH and temperature, the improved retention observed in NaDES-based systems becomes particularly significant [53]. These results clearly demonstrate the NaDES system’s superiority in terms of pigment stability compared to the conventional aqueous extraction method.

Both systems were subjected to comparable thermal processing conditions; however, the NaDES extract retained 40–60% more anthocyanins per gram than the aqueous system. This higher retention likely reflects the stabilizing environment provided by the NaDES. The supramolecular network of hydrogen bonds and relatively low water activity in NaDES may stabilize anthocyanins in the form of flavilium cations while reducing degradation pathways, such as hydration and oxidative reactions [13,54,55]. Given that anthocyanins are well-known antioxidants capable of scavenging reactive oxygen species (ROS), their preservation is particularly relevant for maintaining the potential health benefits associated with reducing oxidative stress, including processes linked to age-related conditions such as sarcopenia [56]. Consequently, the NaDES-based matrix is likely to create an environment that minimizes pigment degradation during the heating and cooling stages of preparing a gummy-type matrix.

The calculated retention efficiency values were low in absolute terms, consistent with previous reports indicating common anthocyanin losses exceeding 90% during gelling and storage in high-moisture jams [57,58]. Despite minor numerical differences, these data reveal consistent trends across all formulations. Samples with higher extract content and a higher proportion of pectin (F1, F5, and F7) exhibited greater anthocyanin retention than samples with a high gelatin content (F3 and F4). These results support the hypothesis that pectin-rich systems enhance anthocyanin trapping and color stability via electrostatic interactions.

The total anthocyanin content of the developed gummies ranged from 0.10 to 0.37 mg C3G/100 g. Direct comparisons with other studies should be interpreted with caution due to differences in plant source, extraction procedures, formulation composition, and analytical methods. Nevertheless, the observed anthocyanin levels indicate that a fraction of these compounds was retained after gummy processing. Variations in anthocyanin retention may be associated with their well-documented sensitivity to thermal treatment, oxygen exposure, and interactions with food matrix components. In addition, interactions between phenolic compounds and gelatin networks may influence the stability and extractability of bioactive molecules within gummy-type systems. These factors collectively contribute to the anthocyanin losses commonly reported during the manufacture and storage of functional confectionery products [58,59,60].

NaDES system showed clear advantages in preserving anthocyanins, suggesting its potential as an eco-friendly solvent for extracting bioactive compounds from grape pomace to develop functional, gummy-like matrices. However, both systems exhibited similarly low overall retention efficiencies.

Table 2 shows the contrasting antioxidant performance of NaDES-based and aqueous gummy formulations, presenting the range of values obtained across the seven formulations. Both systems experienced substantial losses in radical scavenging and reducing power following thermal processing; however, the NaDES-derived gummy matrices retained notably higher absolute values of DPPH, FRAP, and TAC. This difference reflects the greater extraction efficiency of the eutectic solvent of choline chloride and citric acid and its protective capacity against pigment oxidation and polyphenol degradation during formulation.

Interestingly, despite the differences in absolute antioxidant content, the relative retention percentages among the systems remained within a similar range (approximately 0.2–0.4%), suggesting that degradation during gelation and cooling is primarily influenced by the physicochemical properties of the hydrocolloid matrix rather than the solvent system alone. This behavior underscores the pivotal role of the balance between gelatin and pectin, extract concentration, and processing conditions in preserving bioactive compounds.

#### 2.4.3. Antioxidant Activity (DPPH and FRAP) of NaDES- and Aqueous-Based Gummies

The antioxidant activity of grape pomace extracts, as measured by DPPH and FRAP assays, was significantly higher in the NaDES system (4077 mg TE/100 g and 4635 µmol TE/100 g, respectively) than in the aqueous extract (1104 mg TE/100 g and 389.61 µmol TE/100 g). When these extracts were incorporated into gummy-type matrix formulations, both systems exhibited a substantial decrease in antioxidant capacity, which is consistent with previous losses observed for total phenols (TPC) and anthocyanins (TAC).

The estimated DPPH values for the NaDES-based gummy-type matrices ranged from 5 to 19 mg TE/100 g, while the FRAP values ranged from 6.5 to 23 µmol TE/100 g. Gummy-type matrices with an aqueous extract, on the other hand, showed considerably lower antioxidant activity, with DPPH values ranging from 1.2 to 4.8 mg TE/100 g and FRAP values ranging from 0.6 to 2.1 µmol TE/100 g. These results confirm that the NaDES matrix stabilizes redox-active compounds more effectively, likely due to the strong hydrogen-bonding capacity of the choline chloride—citric acid system. This system protects polyphenols from oxidation and metal-catalyzed degradation [61,62,63].

The antioxidant retention pattern in the formulation mirrors that of the TAC. F1 and F7, which have higher extract loading and favorable pectin-to-gelatin ratios, have the highest antioxidant activity, up to 0.46% retention. In contrast, F4 exhibited the weakest activity (<0.15%) due to its low extract content and average gelation density. These results support the hypothesis that pectin-rich networks provide microdomains that can trap hydrophilic antioxidants and that gelatin promotes binding interactions that decrease the extraction capacity and accessibility of phenolic compounds [57].

Although the overall retention of antioxidants is numerically low (0.1–0.5%), it is consistent with other studies that have reported similar losses during the thermal and textural processing of hydrocolloid-based confectionery. The decrease in antioxidant activity, as measured by the DPPH and FRAP assays, can be attributed, in part, to structural modifications induced by heat treatment. Heating promotes the cross-linking of hydrocolloid matrix polymers and facilitates interactions between polysaccharides and phenolic compounds. These interactions may result in the formation of phenolic-polysaccharide complexes or generate non-extractable polyphenols, which reduces their solubility and accessibility during analytical extraction. Consequently, a decrease in in vitro antioxidant activity is observed, though some phenolic compounds remain retained in the food matrix [64,65,66,67]. Similarly, Aiello et al. (2024) [58] found that the remaining polyphenols contributed to the sensory quality and functional labeling of gummy candies despite significant thermal degradation.

Comparing the two systems revealed that the gummy-type matrices prepared with NaDES retained approximately four times more antioxidant activity than their aqueous counterparts. This reflects the combined effects of higher extraction efficiency, pigment stabilization, and reduced oxygen reactivity. However, both systems exhibited similar retention rate trends, suggesting that antioxidant loss primarily occurs during the heating and dehydration stages of gummy matrix formulation rather than during extraction.

### 2.5. Global Correlation/Selection of the Optimal Formulation

A principal component analysis (PCA) was conducted to investigate the interrelationships among the physicochemical, textural, and bioactive characteristics of the gummy-type matrix formulations. The first two principal components explained 79.6% of the total variance: 54.2% for PC1 and 25.4% for PC2. This indicates that the model adequately represents the multivariate structure of the dataset.

PC1 was primarily associated with positive loadings for elasticity and water activity (a_w_) and negative loadings for hardness, antioxidant activity (DPPH), total polyphenol content (TPC), and moisture. The opposite orientation of elasticity and hardness confirms their antagonistic mechanical behavior within hydrocolloid gel matrices.

Formulations F1, F2, and F4 were in the region associated with higher a_w_ and elasticity, suggesting less compact gel structures with greater water mobility. In contrast, formulations F6 and F7 were located in the negative region of PC1, which correlates with higher hardness and antioxidant activity. This could potentially reflect greater structural density and stronger interactions between the hydrocolloid matrix and the bioactive compounds.

PC2 primarily distinguished between formulations with higher total phenolic content (TPC) and moisture content (positive loadings) and those with higher DPPH and hardness (negative loadings). Formulation F5 showed a strong association with TPC and moisture content. Meanwhile, F3 and F7 were linked to higher firmness and antioxidant activity. These results suggest that total phenolic content and antioxidant activity do not respond identically; rather, they depend on the nature and availability of the compounds present in each formulation.

### 2.6. Identification of the Most Balanced Formulation

**Figure 4:**
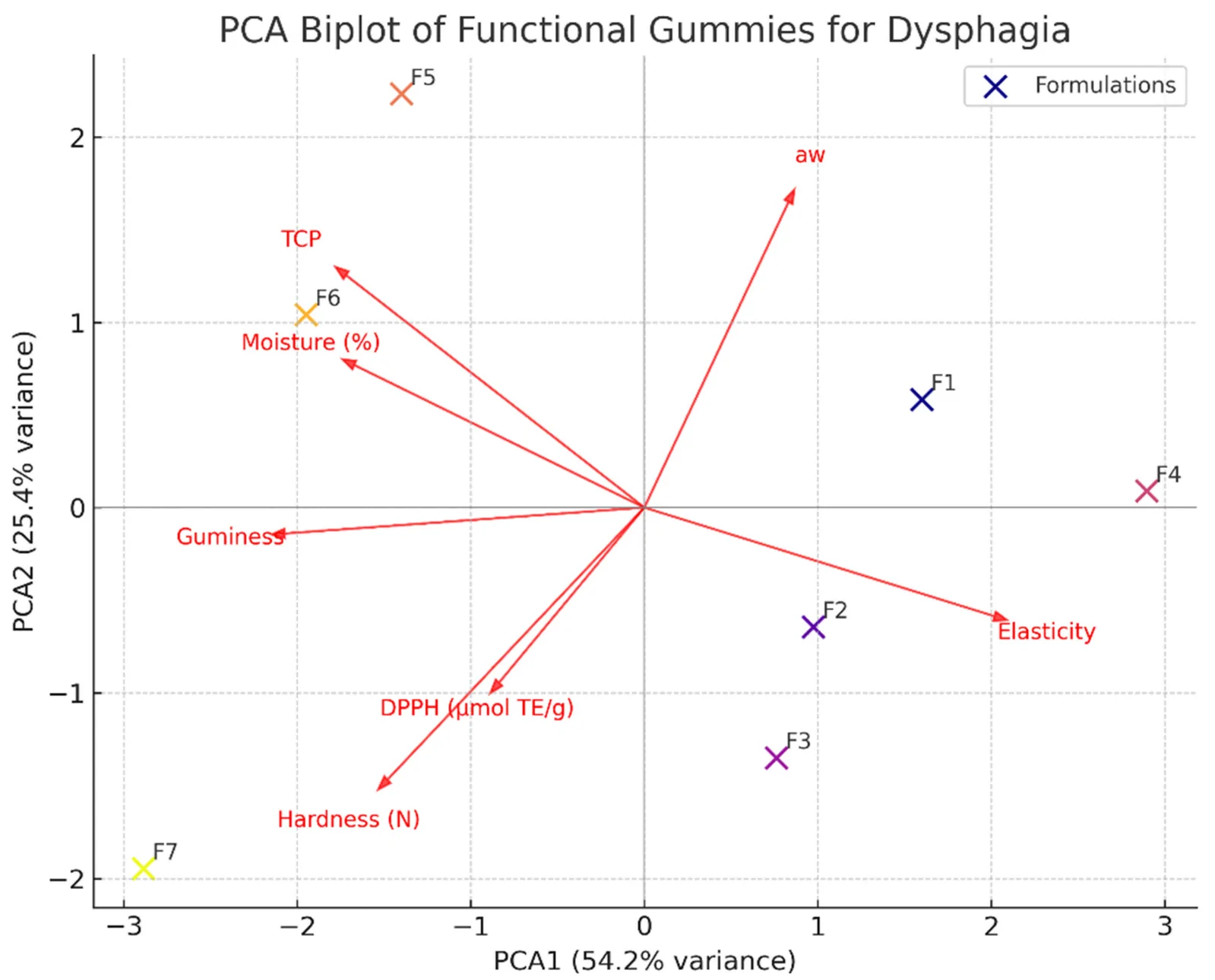
Biplot of the Principal Component Analysis (PCA) of the formulations of functional gummies for dysphagia. PC1 explained 54.2% and PC2 explained 25.4% of the total variance (79.6% cumulative).

As shown in Figure 4, the first two principal components explained 79.6% of the total variance (PC1 = 54.2% and PC2 = 25.4%), indicating that the PCA biplot adequately summarized the multidimensional relationships among the formulations and the evaluated physicochemical, textural, and functional variables. The high cumulative variance explained confirms that the ordination captured most of the relevant information contained in the dataset and provides a reliable basis for interpreting formulation behavior.

The distribution of the loading vectors revealed two major trends within the experimental space. Total phenolic content (TPC) and antioxidant activity were closely aligned, confirming the strong positive relationship between phenolic retention and antioxidant capacity. In contrast, hardness and gumminess were projected in a different direction, indicating that increasing gel strength was not necessarily accompanied by improved functional performance. Moisture content occupied an intermediate position, suggesting its dual contribution to maintaining hydrogel hydration while simultaneously influencing the mechanical properties of the polymeric network.

The separation of the formulations along PC1 primarily reflected the balance between structural resistance and functional quality, whereas PC2 further discriminated samples according to differences in water retention and secondary textural responses. Together, these components distinguished formulations exhibiting dense and mechanically resistant gel networks from those characterized by greater preservation of phenolic compounds and antioxidant activity.

Among all formulations, F5 occupied a central position within the PCA space, exhibiting simultaneous proximity to the vectors associated with TPC, antioxidant activity, moderate moisture content, and intermediate textural parameters. Rather than representing a simple intermediate formulation, this location reflects a multivariate equilibrium in which no single quality attribute predominated over the others. The balanced position of F5 indicates that the selected hydrocolloid composition successfully reconciled the competing requirements of gel firmness, water retention, and bioactive preservation.

This multivariate behavior is particularly noteworthy when interpreted in the context of the mixture design. Because the Simplex-Centroid design systematically explores the interactions between gelatin and low-methoxyl pectin, the central location of F5 suggests that its composition lies within a synergistic region of the response surface rather than at an extreme dominated by either hydrocolloid. At this composition (6% gelatin and 1% pectin), gelatin provides an elastic protein network that contributes structural cohesion, while pectin enhances water-binding capacity and reinforces network stability. The resulting matrix appears capable of efficiently incorporating the NaDES extract while limiting the loss or degradation of phenolic compounds during gel formation. Consequently, the formulation achieves a favorable balance between network cohesion and molecular mobility, allowing the retention of bioactive compounds without generating an excessively rigid structure.

The position of F5 between the clusters defined by mechanical resistance and functional attributes also reflects the inherent trade-off characteristic of hydrogel-based food systems. Increasing polymer interactions generally promotes higher hardness and gumminess, improving dimensional stability but simultaneously reducing deformability. Conversely, softer gels facilitate oral processing but may compromise structural integrity and bioactive retention. F5 appears to overcome this trade-off by maintaining sufficient mechanical strength for handling and storage while preserving the deformability required for efficient bolus formation and safe swallowing.

In contrast, formulations such as F7 were displaced toward the region associated with greater hardness and gumminess, indicating the formation of denser gel networks with higher resistance to deformation. Although these characteristics may improve mechanical stability, they are less desirable for dysphagia-oriented products because they increase the force required during oral processing. Conversely, formulations located closer to the moisture vector but farther from the hardness vector exhibited softer textures but comparatively lower structural resistance, illustrating that excessive water retention alone is insufficient to optimize overall product quality.

Overall, the PCA integrates the physicochemical, textural, and functional datasets into a single multivariate framework and demonstrates that formulation performance is governed by the interaction among hydrocolloid composition, moisture distribution, and bioactive compound retention rather than by any individual variable. The agreement between the PCA and the individual response analyses validates the optimization strategy derived from the mixture design and identifies F5 (6% gelatin, 1% pectin, and 7.5% NaDES extract) as the formulation providing the most favorable combination of structural stability, phenolic preservation, antioxidant capacity, and textural characteristics compatible with safe swallowing. These results reinforce the effectiveness of the Simplex-Centroid approach for identifying optimal hydrocolloid proportions in the development of functional gummy-type foods intended for individuals with presbyphagia.

### 2.7. Future Research Directions

Future research should focus on evaluating the sensory attributes of the developed gummy formulations, as these factors are critical for their successful implementation as functional food products. In addition, storage studies are needed to determine the stability of key quality parameters, including polyphenol content, antioxidant activity, color, texture, and moisture-related properties throughout the product shelf life. Given the relatively high moisture content of gummy matrices, future work should also include microbiological assessments to ensure product safety and establish appropriate storage conditions and shelf-life limits. Furthermore, although the formulations were designed to comply with the textural requirements for individuals with presbyphagia, future studies should incorporate practical IDDSI testing together with in vivo swallowing assessments and clinical acceptability studies to confirm their safety, swallowing performance, and suitability for the target population under real consumption conditions. These investigations will provide essential information regarding the technological feasibility, stability, safety, clinical applicability, and consumer acceptance of the developed formulations.

## 3. Conclusions

This study evaluated the influence of gelatin–pectin ratio and extraction medium on the physicochemical, textural and bioactive properties of gummy-type matrices enriched with País grape pomace extracts. The use of NaDES as extraction medium resulted in higher retention of total phenolics, anthocyanins and antioxidant activity compared with aqueous extraction. Matrix composition also significantly affected hardness, elasticity and moisture-related properties, demonstrating the importance of hydrocolloid balance in determining product characteristics.

Of all formulations evaluated, F5 (6% gelatin, 1% pectin, and 7.5% NaDES extract) exhibited the highest total polyphenol content and balanced antioxidant activity, alongside a texture profile compatible with IDDSI levels 5–6, making it ideal for individuals with mild dysphagia. Multivariate analysis further confirmed F5’s central position within the design space, reflecting equilibrated interactions among water availability, structural properties, and bioactive retention.

These findings support the potential application of NaDES-extracted grape pomace ingredients in hydrocolloid-based food matrices. However, further studies are required to evaluate sensory acceptance, storage stability and shelf life.

## 4. Materials and Methods

Grape pomace (*Vitis vinifera* L., “País” variety) was supplied by local producers in the Itata Valley (Ñuble, Chile), specifically from the municipality of Quirihue, between April and May 2024; it was frozen on the same day it was received at −20 °C. Subsequently, the pomace was dried at 50 °C in an oven (Memmert GmbH + Co. KG, Schwabach, Germany), ground, and sieved to obtain particles with a diameter of 0.5 mm.

To obtain grape extracts, ultrasonic-assisted extraction was performed using a Bransson Ultrasonic MTCPX1800H-E device (Danbury, CT, USA) operating at 40 kHz. The solvents used were choline chloride and citric acid (ChCl-CA) and water to obtained both extracts. The ratio of raw material to solvent was 1 g of raw material per 8 mL of solvent. The bath temperature was 60 °C using the temperature control system integrated in the ultrasonic equipment. The samples were then centrifuged, and the resulting supernatant was stored at 4 °C.

### 4.1. Experimental Design

A three-factor Simplex-Centroid mixture design was applied to evaluate the combined effect of gelatin, pectin, and plant extract proportions on the physicochemical properties of the formulated gummy system. The proportions of the three mixture components were systematically varied according to a seven-run experimental matrix (Table 3), whereas the remaining formulation ingredients (isomalt, guar gum, calcium lactate (2% of the weight of the pectin-dependent ratio), and water) were maintained under fixed formulation conditions to minimize their influence on the evaluated responses.

The Simplex-Centroid design comprised three vertex formulations corresponding to mixtures enriched in each individual component, three binary formulations representing equal combinations of two components, and one centroid formulation containing balanced contributions from all three components. This configuration enables the estimation of both individual and interaction effects of the mixture variables while requiring a limited number of experimental runs.

The jelly formulations were prepared by first hydrating the gelatin in distilled water at room temperature for 8–12 h to ensure complete swelling. Separately, a 70% (*w*/*w*) isomalt syrup was prepared by heating a mixture of isomalt and water to approximately 90 °C under constant stirring (450 rpm). Citrus pectin was gradually added in dry form to the hot syrup and mixed until completely dispersed and dissolved. Next, the pre-hydrated gelatin was added under continuous stirring (450 rpm) until a homogeneous mixture was obtained. Subsequently, calcium lactate was added under the same stirring conditions until completely dissolved. The mixture was cooled to a temperature not exceeding 60 °C, after which the País grape pomace extract (aqueous extract or NaDES-derived extract, depending on the formulation) was added to minimize the thermal degradation of the phenolic compounds. Guar gum was then gradually added in dry form under continuous stirring (450 rpm) until completely homogenized. The resulting gel-like mass was immediately poured, while still hot, into silicone molds, allowed to gel in a refrigerator (4 ± 1 °C) for 24 h.

After demolding, the gummies were immersed in a 2% (*w*/*v*) corn starch suspension to obtain a thin surface coating that reduced stickiness and facilitated handling during the drying process. The starch concentration was selected to minimize surface tackiness while avoiding excessive opacity or visible alteration of the gummy appearance. After coating, excess starch suspension was removed, and the gummies were dried in a forced-air oven at 50 °C until constant weight prior to physicochemical, textural, color, and antioxidant analyses. All formulations were prepared under identical processing conditions, and the experimental responses were analyzed using response surface methodology (RSM) to identify the optimal formulation within the investigated design space.

Food-grade bovine gelatin (240 Bloom; Gelnex^®^, Itá, Brazil) and citrus pectin (Guttche^®^, food grade, Concepción, Chile) were selected as the gelling agents because their complementary gelation mechanisms enable the formation of stable three-dimensional networks with desirable textural properties. Gelatin contributes elasticity and cohesiveness, whereas pectin enhances firmness, water-holding capacity, and resistance to deformation. Such composite hydrocolloid systems have been widely employed in functional gummy formulations because they facilitate the incorporation of plant-derived bioactive compounds while maintaining product integrity and consumer-acceptable texture. Furthermore, the interaction between both polymers can contribute to limiting moisture migration and improving the physical stability of the final product during storage [41,44,68].

### 4.2. Physical and Chemical Properties

#### 4.2.1. Water Activity (a_w_)

An AquaLab 4TE water activity meter (Meter Group Inc., Pullman, WA, USA) was used to determine the water activity (a_w_) of the samples. Approximately 4 g of the sample was placed in the measurement capsule and equilibrated at 20 ± 1 °C until a stable reading was obtained [69,70]. The results were expressed as dimensionless a_w_ values. All measurements were performed in triplicate.

#### 4.2.2. Moisture Content

Moisture content was determined using the gravimetric oven-drying method. Approximately 10 g of sample was weighed into pre-dried and tared capsules, which were then placed in a Memmert UN55 oven (Memmert GmbH + Co. KG, Schwabach, Germany) with a 55 L capacity at 105 °C until a constant weight was reached. Analyses were performed in triplicate.

#### 4.2.3. Color

The instrumental color of the samples was determined using a Konica Minolta CM-5 spectrophotometer (Konica Minolta, Osaka, Japan) that had been previously calibrated with a white standard. Measurements were taken using the CIELab system, yielding the following parameters:

L* (luminosity);

a* (red–green coordinate);

b* (yellow-blue coordinate).

Measurements were taken at various points on each sample’s surface, and the results were expressed as an average of three measurements.

The total color difference (ΔE) was calculated using the following equation:

(1) $$\Delta E\;=\;\sqrt{{\left(L^{*}\;-\;L_{0}^{*}\right)}^{2}+{\left(a^{*}\;-\;a_{0}^{*}\right)}^{2}+{\left(b^{*}\;-\;b_{0}^{*}\right)}^{2}}$$

where

L*,a*, b* correspond to the sample values.

L_0_*,a_0_*,b_0_* correspond to the values of the reference or control sample.

The color intensity or saturation (C*ab) was calculated using the equation:

(2) $$C^{*}ab\;=\;\sqrt{{\left(a^{*}\;\right)}^{2}+{\left(b^{*}\right)}^{2}}$$

#### 4.2.4. Textural Properties

The samples’ textural properties were evaluated using Texture Profile Analysis (TPA) with a TA.XTPlus 100 texture analyzer (Stable Micro Systems Ltd., Godalming, UK). Texture profiling is a widely used method for characterizing the mechanical properties of foods because it allows for the quantification of instrumental attributes related to sensory perception of texture [20,71].

This method involves a double compression test that simulates the chewing process and allows for the determination of various mechanical parameters associated with food texture [20].

During the test, the samples underwent two consecutive compression cycles using an appropriate probe for the sample type. All measurements were performed in triplicate, and the following textural parameters were determined from the resulting force-time curves:

1. Hardness: maximum force recorded during the first compression.
2. Cohesiveness: ratio of the area under the curve of the second compression to that of the first.
3. Springiness: the sample’s ability to return to its original shape after being deformed.
4. Chewiness or gumminess when applicable.

### 4.3. Bioactive Properties

It was determined using the Folin–Ciocalteu method, according to the methodology of Kuskoski et al. (2005) [72]. For the reaction, 0.25 mL of extract, 1.25 mL of Folin–Ciocalteu reagent, and 2.5 mL of 20% Na_2_CO_3_ aqueous solution were used. The solution was left in the dark. The solution was left in a dark place and after two hours of reaction, the absorbance reading was taken at 765 nm in a spectrophotometer (UV-VIS, PG T-70 Spectrometer; Greenville, SC, USA). A calibration curve was elaborated with a gallic acid standard using solutions of 20, 40, 60, 80, and 100 ppm to express the results in mg of gallic acid. The results were expressed in mg of gallic acid equivalent (GAE)/100 g of dry sample.

The total anthocyanin content was determined using the methodology described by Vidal-San Martín (2021) [53], quantified using the pH differential method. First, a 400 μL sample was added to 3600 μL of potassium chloride (KCl, pH 1.0, 0.025 M) and 3600 μL of sodium acetate (CH_3_COONa, pH 4.5, 0.4 M). Absorbance was measured at wavelengths of 510 and 700 nm using a UV-visible spectrometer (PG T-70; Greenville, SC, USA). The differential absorbance was calculated using the following equation:

∆A = (A_510_ − A_700_)pH 1.0 − (A_510_ − A_700_)pH 4.5 (3)

Anthocyanin (mg/mL) = (∆A × MW × FD × 1000)/(ꜫ × 1)

where ∆A is the absorbance difference at pH 1 and 4.5, FD is the dilution factor, ε is the molar extinction coefficient (29,000) of delphinidin-3-glucoside, and MW is the molecular weight of delphinidin-3-glucoside (462.5 g/mol). The results are expressed in mg C3G/100 g.

The antioxidant capacity was determined using the DPPH (2,2-diphenyl-1-picrylhydrazyl) assay [73], and by measuring plasma ferric reduction capacity (FRAP). The assay was performed according to the modified methodology of [74]. Results were expressed as milligrams of Trolox equivalents per milliliter of sample (mg TE/mL) and micromoles of Fe^2+^ per 100 g or milliliters of sample, respectively.

Recovery (or anthocyanin retention) was estimated using a mass-balance approach, in which the theoretical concentration of each bioactive compound in the gummies was calculated from the concentration measured in the corresponding liquid extract and the proportion of extract incorporated into each formulation (5.0–10.0%, *w*/*w*). The experimental concentration measured in the gummies was then expressed as a percentage of this theoretical value, following the general concept of retention factors described for food processing studies.

(4) $$Recovery\left(\%\right)=\frac{C_{gummy}}{C_{extract}\times (E/100)}\times 100$$

where:

C_gummy_ = experimental concentration in the gummy;

C_extract_ = concentration measured in the liquid extract;

E = percentage of incorporated extract (5, 7.5 o 10%, *p*/*p*).

### 4.4. Statistical Analysis

The data were analyzed using Statgraphics Centurion XVI software (Statistical Graphics Corp., Herndon, VA, USA). All experiments were conducted in triplicate, with three independent samples included in each replicate. Significant differences among treatments were assessed using Fisher’s least significant difference (LSD) test at 95% confidence level. Additionally, principal component analysis (PCA) was performed to evaluate the correlations among the variables, as well as the variability associated with the interactions between the soil properties and the antioxidant activity of the obtained extracts.

## Figures and Tables

**Table 1:** Colorimetric parameters (L, a, b*, ΔE, and C*ab) of gummy formulations prepared with NaDES and aqueous País grape extract.

	NaDES Formulation					Aqueous Formulation					
Runs	L*	a*	b*	ΔE	C*ab	Runs	L*	a*	b*	ΔE	C*ab
**F1**	31.08 ± 1.16 ^a^	6.54 ± 1.02 ^a^	5.82 ± 0.56 ^ab^	32.32 ± 0.82 ^ab^	8.76	**F1**	29.71 ± 0.13 ^b^	6.72 ± 0.20 ^ab^	4.63 ± 0.07 ^a^	20.56 ± 0.81 ^a^	8.16
**F2**	32.46 ± 0.13 ^a^	10.89 ± 0.20 ^b^	5.54 ± 0.17 ^ab^	34.68 ± 0.16 ^a^	12.23	**F2**	31.12 ± 0.75 ^a^	5.89 ± 0.19 ^b^	1.46 ± 0.32 ^b^	32.69 ± 0.71 ^b^	6.07
**F3**	27.77 ± 1.02 ^b^	12.45 ± 2.90 ^b^	4.21 ± 1.27 ^a^	30.76 ± 2.31 ^b^	13.09	**F3**	27.77 ± 0.24 ^c^	8.93 ± 0.79 ^c^	2.48 ± 0.07 ^cd^	29.28 ± 0.46 ^ac^	9.27
**F4**	32.41 ± 2.11 ^ac^	10.91 ± 1.06 ^b^	7.04 ± 1.21 ^b^	34.91 ± 2.52 ^a^	12.98	**F4**	30.65 ± 0.58 ^ab^	6.60 ± 0.49 ^ab^	2.47 ± 0.51 ^cd^	31.45 ± 0.67 ^ab^	7.05
**F5**	30.63 ± 0.70 ^cd^	12.18 ± 0.84 ^b^	5.92 ± 0.10 ^ab^	33.49 ± 0.95 ^ab^	13.54	**F5**	25.72 ± 0.53 ^d^	7.84 ± 0.06 ^ac^	2.37 ± 0.16 ^c^	27.00 ± 0.51 ^d^	8.19
**F6**	31.54 ± 0.14 ^ac^	9.55 ± 0.40 ^ab^	5.03 ± 1.34 ^ab^	33.35 ± 0.18 ^ab^	10.79	**F6**	24.93 ± 1.09 ^d^	7.19 ± 0.48 ^a^	1.99 ± 0.36 ^bc^	26.03 ± 0.91 ^d^	7.46
**F7**	27.23 ± 0.69 ^bd^	11.24 ± 0.74 ^b^	3.73 ± 0.71 ^a^	29.71 ± 0.28 ^b^	11.84	**F7**	27.68 ± 0.75 ^c^	8.90 ± 0.51 ^c^	3.18 ± 0.26 ^d^	29.28 ± 0.61 ^c^	9.54

Note: Details of the nomenclature and the corresponding formulations are presented in Table 3. Different lowercase letters indicate statistically significant differences (*p* < 0.05).

**Table 2:** Comparative recovery and retention of antioxidants from grape pomace in NaDES-based and aqueous gummy bean-type product formulations.

Parameter	Initial Extract (NaDES)	Initial Extract (Aqueous)	Gummies (NaDES, F1–F7)	Gummies (Aqueous, F1–F7)	Main Observation
**TPC (mg GAE/100 g)**	1053.57	288.99	58.04–89.43	44.09–70.98	Higher in NaDES formulations
**TAC (mg C3G/100 g)**	77.49	44.08	0.100–0.367	0.055–0.202	Higher pigment preservation with NaDES
**DPPH (mg TE/100 g)**	4077	1104	5.0–19.0	1.2–4.8	Higher antioxidant activity with NaDES
**FRAP (µmol TE/100 g)**	4635	389.61	6.5–23.0	0.6–2.1	Greater reducing power with NaDES
**Anthocyanin retention (%)**	—	—	1.52–5.30	2.58–8.62	Similar relative retention

**Table 3:** Experimental design matrix showing the proportions of gelatin, pectin and extract used in formulations F1–F7.

Runs	Gelatin (G) %	Pectin (P) %	Extract (E) %	Isomalt Syrup	Guar Gum	Calcium Lactate	Water
F1	8	1	5	35	0.2	0.02	50.78
F2	4	3	5	35	0.2	0.06	52.74
F3	4	1	10	35	0.2	0.02	49.78
F4	6	2	5	35	0.2	0.04	51.76
F5	6	1	7.5	35	0.2	0.02	50.28
F6	4	2	7.5	35	0.2	0.04	51.26
F7	6	3	10	35	0.2	0.06	45.74

## Data Availability

The data presented in this study are available from the corresponding author upon reasonable request.
